# Super-delta: a new differential gene expression analysis procedure with robust data normalization

**DOI:** 10.1186/s12859-017-1992-2

**Published:** 2017-12-21

**Authors:** Yuhang Liu, Jinfeng Zhang, Xing Qiu

**Affiliations:** 1 Department of Statistics, Florida State University, Tallahassee, 32306 FL USA; 2 Department of Biostatistics and Computational Biology, University of Rochester, Rochester, 14624 NY USA

**Keywords:** Gene expression, Differential expression analysis, Super-delta, Robust normalization, Modified *t*-test

## Abstract

**Background:**

Normalization is an important data preparation step in gene expression analyses, designed to remove various systematic noise. Sample variance is greatly reduced after normalization, hence the power of subsequent statistical analyses is likely to increase. On the other hand, variance reduction is made possible by borrowing information across all genes, including differentially expressed genes (DEGs) and outliers, which will inevitably introduce some bias. This bias typically inflates type I error; and can reduce statistical power in certain situations. In this study we propose a new differential expression analysis pipeline, dubbed as super-delta, that consists of a multivariate extension of the global normalization and a modified *t*-test. A robust procedure is designed to minimize the bias introduced by DEGs in the normalization step. The modified *t*-test is derived based on asymptotic theory for hypothesis testing that suitably pairs with the proposed robust normalization.

**Results:**

We first compared super-delta with four commonly used normalization methods: global, median-IQR, quantile, and cyclic loess normalization in simulation studies. Super-delta was shown to have better statistical power with tighter control of type I error rate than its competitors. In many cases, the performance of super-delta is close to that of an oracle test in which datasets without technical noise were used. We then applied all methods to a collection of gene expression datasets on breast cancer patients who received neoadjuvant chemotherapy. While there is a substantial overlap of the DEGs identified by all of them, super-delta were able to identify comparatively more DEGs than its competitors. Downstream gene set enrichment analysis confirmed that all these methods selected largely consistent pathways. Detailed investigations on the relatively small differences showed that pathways identified by super-delta have better connections to breast cancer than other methods.

**Conclusions:**

As a new pipeline, super-delta provides new insights to the area of differential gene expression analysis. Solid theoretical foundation supports its asymptotic unbiasedness and technical noise-free properties. Implementation on real and simulated datasets demonstrates its decent performance compared with state-of-art procedures. It also has the potential of expansion to be incorporated with other data type and/or more general between-group comparison problems.

**Electronic supplementary material:**

The online version of this article (doi:10.1186/s12859-017-1992-2) contains supplementary material, which is available to authorized users.

## Background

Gene expression data analysis has become a popular research area in the 21st century. To identify differentially expressed (DE) genes, namely those that have significantly different mean expression levels between groups of samples (e.g., cancer patients and normal people), is one of the most common tasks in transcriptome research.

Due to the complex nature of high-throughput expression data, true gene expression levels that may be associated with the underlying biological conditions are almost always confounded by sample-specific variation induced by various technical noise and batch effects. Unlike common *i*.*i*.*d*. random measurement error pertain to a specific gene for a subject, sample-specific variation affects thousands of genes at a time and can increase both variance and inter-gene correlation significantly. Over the past two decades, several normalization procedures have been proposed to remove sample-specific variation for microarray data. The first approach [1, 2] depends on the use of a small subset of genes (housekeeping genes) that are expected to have *constant* true gene expression levels for all samples. In other words, housekeeping genes are assumed to be not differentially expressed (NDE) a priori. Based on the constant expression assumption, the *observed* variation of housekeeping genes must be a combination of sample-specific and *i*.*i*.*d*. noise. By the strong law of large numbers, the *i*.*i*.*d*. noise tend to get cancelled out when we average a relatively large number of such housekeeping genes, so the remaining variation can be considered as an “estimate” of the sample-specific noise. Subtracting these estimates from the transcriptome reduces noise and spurious correlation, and makes samples more comparable for subsequent statistical analyses. The main drawback of this approach is that the set of “housekeeping” gene is a moving target that depends on the biological conditions and can hardly be made objective. Furthermore, even if the mean expression of a housekeeping gene is constant for all conditions in a given study, it may exhibit natural variability for different subjects [3] that are disadvantageous for normalization. The second approach is to use objective, data-driven methods to transform data so that certain statistical characteristics are made constant for all normalized samples in a pool. Popular choices include the global normalization [2, 4] that makes (trimmed) mean of normalized samples identical; median-IQR normalization [5] that makes both location (median) and scale (IQR) identical; and the quantile normalization [6] that aligns the entire sample distribution curves across all samples. These normalization methods do not depend on the somewhat subjective selection of housekeeping genes and are much more popular in current practice than the first approach. By and large, all normalization methods tend to reduce both sample variation and inter-gene correlation sharply and make the analytical results more stable, if not always more powerful [7–9]. Because the second type of normalization achieves variance-reduction by borrowing information across *all* genes (and samples, in the quantile normalization case) irrespective of whether these genes are housekeeping or not, they also introduce certain bias that may reduce statistical power [8] and/or inflate type I error rate [7] when a non-trivial proportion (e.g., more than 10%) of all genes are DE and the differential expression pattern is not balanced, namely number of up-regulated genes does not equal that of down-regulated genes. A data-driven variable transformation called the *δ*-sequence method [10, 11] is an alternative to the aforementioned normalization procedures. In this method, every gene is normalized by another one with similar variance that acts as the housekeeping gene. Unlike standard normalization procedures that use the same sample means/medians/quantiles as the references for every gene, *δ*-sequence method selects one *specific* housekeeping gene for each gene. The theoretical considerations of this approach is explained in [12]. As a consequence, the *δ*-sequence method is a *local* normalization method because only one gene is needed to normalize a given gene. This property is especially important for translating results from whole-transcriptome analyses to clinical applications in which only a dozen or so genes will be used as *biomarkers* for biological conditions such as a specific type of cancer – we no longer have the luxury of borrowing information from thousands of genes for normalization. Simulation studies showed that compared with competing methods, *δ*-sequence is more robust for studies with unbalanced expression patterns but is more likely to be under-powered and always has a “fixed” type I error resulted from imperfect “pair-breaking” procedure [7]. These disadvantages make *δ*-sequence method only applicable for studies with very large (e.g., $n \geqslant 100$) sample size and relatively strong signal.

In this study, we propose a new gene expression analysis pipeline for microarray platforms called super-delta that consists of a data-driven housekeeping gene identification step and a modified DE gene selection step. In the first step, one pairing housekeeping gene is identified for each gene based on a robust statistical method. In the second step, DE genes are selected based on a modified two-sample *t*-test that accommodates for the unique distributional properties of the normalized expressions. Based on large-sample theory, we demonstrated that up to an *O*(*n* ^−1/2^) term, our pipeline is asymptotically equivalent to applying *t*-tests to a hypothetical expression data free of sample-specific noise (dubbed as the oracle test in our study).

In simulation studies, we compared super-delta with four popular competing normalization methods: global, medIQR, LOESS, and quantile. We think these four methods represent a spectrum of normalization methods that range from “very parametric” (global) to “very nonparametric” (quantile). We expect the performance of other methods, such as a variant of global normalization with trimmed mean or median, can be represented as members within this spectrum. Our simulation studies showed that super-delta almost always has higher statistical power and lower type I error rate than the competing methods. In fact, its statistical performance is very close to that of the oracle test.

Finally, we applied super-delta and competing methods to a large-scale dataset with 242 breast cancer patients who received neoadjuvant chemotherapy. We found that while the overlap of DE genes identified by all five methods is very high, super-delta always identified more DE genes. Literature search confirmed that these unique DE genes are known to have biological connections to breast cancer or chemotherapy. Functional enrichment analyses based on these DE genes confirmed high consistency of all methods at the pathway level; and super-delta identified two unique pathways that are intricately involved in breast cancer.

## Methods

### Gene expression data from breast cancer patients

Gene expression data collected from 242 breast cancer patients who took Docetaxel and Anthracycline (TxA) chemotherapy are used in this study. Among them, 80 patients are identified as pathologic complete response (pCR, or group *A*) and 162 are identified as residual disease (RD, or group *B*) based on their clinical responses. More specifically, we download the raw gene expression files in .CEL format from Gene Expression Omnibus (GEO, [13]) series GSE20194 [14], GSE23988 [15], GSE25065 [16], and GSE42822 [17]. All data are summarized by the robust multi-array average method [6, 18, 19]. After log2 transformation and non-specific filtering that removed 50% of genes with low inter-quartile range (IQR) to the data, expression levels of 11,141 probe sets are reported for each sample. Throughout this manuscript, we denote the log-transformed, un-normalized expression level of the *i*th gene sampled from the *j*th subject as $y^{a}_{ij}$, where *a*=*A*,*B* is the group to which the *j*th subject belong.

### Normalization methods

In this study, we compare the performance of super-delta with four commonly used normalization methods, global, medIQR, quantile, and loess. We briefly introduce these methods as follows.

- Global normalization (global). Let $\bar {y}^{a}_{j}=\frac {1}{m}\sum _{i=1}^{m} y^{a}_{ij}$, *j*=1,2,…,*n*, *a*=*A*,*B*, be the mean expression of sample *j*. Then the normalized expression is defined as $y^{a,*}_{ij} = y^{a}_{ij} - \bar {y}^{a}_{j}$; *i*=1,2,…,*m*, *j*=1,2,…,*n*, *a*=*A*,*B*. The idea of global normalization is simple and straightforward: It uses the per-sample mean expression as the “housekeeping gene” for all genes. Note that sometimes a constant, such as the overall mean expression across all samples, is added to all $y^{a,*}_{ij}$ to avoid negative values. Clearly, this practice does not affect the subsequent differential expression analysis.
- Median-IQR normalization (medIQR). Let Med_*j*_ and IQR_*j*_, *j*=1,2,…,*n*, be the sample median and IQR computed from all gene expressions in sample *j*. The median of Med_*j*_ and the median of IQR_*j*_ are denoted by Med_0_ and IQR_0_, respectively. For each sample *j*, expressions are adjusted by first subtracting the sample’s median Med_*j*_, and then multiplied by the ratio $\frac {\text {IQR}_{0}}{\text {IQR}_{j}}$. Finally, the median of the medians Med_0_ is added to all values. This procedure is essentially a location-scale transformation based on sample median and IQR, which are more robust to outliers than sample mean and standard deviation. Apparently, normalized samples have the same median (Med_0_) and IQR (IQR_0_).
- Quantile normalization (quant). First, a reference array of empirical quantiles, denoted as **q**=(*q*_1_,*q*_2_,…,*q*_*m*_), is computed by taking the average across all ordered arrays. Let $y^{a}_{(1),j} \leqslant y^{a}_{(2),j} \leqslant \cdots \leqslant y^{a}_{(m),j}$ denote the ordered gene expression observations in the *j*th array (*j*=1,2,…,*n*) of the *a*th (*a*=*A*,*B*) group, the *r*th (*r*=1,2,…,*m*) element of this reference array is
  1 $$  q_{r} = \frac{1}{2n} \left(\sum_{k=1}^{n} y^{A}_{(r),k} + \sum_{l=1}^{n} y^{B}_{(r),l} \right).  $$
  Next, the original expressions are replaced by the entries of the reference array with the same rank. The normalized gene expressions are
  2 $$  y^{a,*}_{ij} = q_{r^{a}_{ij}} = \frac{1}{2n} \left(\sum_{k=1}^{n} y^{A}_{(r^{a}_{ij}),k}+\sum_{l=1}^{n} y^{B}_{(r^{a}_{ij}),l} \right).  $$
  Interested readers can find more details in [6].
- Cyclic loess normalization (loess). This is a between-array normalization method based on removing spurious variation estimated by local regression. This method is described in [6, 20] and implemented in several R packages. For this study, we use the implementation provided by the LIMMA package [21].

### The bias-variance trade-off of the normalization procedures

In this section, we would like to briefly discuss the bias-variance trade-off of normalization procedures based on the following widely adopted mixed effects model for gene expression [7, 22–25]. For genes *i*=1,2,…,*m*; samples *j*=1,2,…,*n*; and two conditions *a*=*A*,*B*, the log2-transformed gene expression level is modeled as

3 $$  \begin{aligned} y^{a}_{ij} &= \mu^{a}_{i} + \epsilon^{a}_{ij} +\alpha^{a}_{j} = x^{a}_{ij} + \alpha^{a}_{j}. \end{aligned}  $$

Here $\mu ^{a}_{i}$ is the mean expression value of the *i*th gene in group *a*; $\alpha ^{a}_{j} \sim N(0, \eta ^{2})$ is a random effect term that represents per-sample variation specific to the *j*th sample; $\epsilon ^{a}_{ij} \sim N(0, \sigma ^{2})$ is an *i*.*i*.*d*. random variable that represents both measurement error and true biological variation that cannot be explained by $\alpha ^{a}_{j}$; $x^{a}_{ij} := \mu ^{a}_{i} + \epsilon ^{a}_{ij}$ can be considered as the *oracle* gene expression level that is free of per-sample variation.

Let us denote the mean group difference by $d_{i} := \mu _{i}^{A}-\mu _{i}^{B}$. We are interested in testing the following hypotheses

4 $$  H_{0}^{(i)}: d_{i} = 0, \quad \text{v.s.} \quad H_{1}^{(i)}: d_{i} \ne 0, \qquad i=1, 2, \ldots, m.  $$

Empirical evidences show that *η* ^2^ is typically much greater than *σ* ^2^ [9], so applying a suitable normalization procedure to reduce the per-sample variation increases the statistical power in most cases, as compared to applying two-group tests to non-normalized data [8]. However, normalization procedures borrow information from both DE and NDE genes to reduce the impact of $\alpha ^{a}_{j}$, so it creates certain bias that may inflate type I error rate and reduce testing power. As an example, based on Eq. (3), global normalized expressions are

5 $$  y^{a,*}_{ij}=y^{a}_{ij} - \bar{y}^{a}_{j} = \left(\mu^{a}_{i} - \bar{\mu}^{a}\right) + \left(\epsilon^{a}_{ij} - \bar{\epsilon}^{a}_{\cdot j} \right),  $$

which is free of $\alpha ^{a}_{j}$ (variance reduction). On the other hand, the expected group difference after global normalization is

6 $$  E \left(y^{A,*}_{ij} - y^{B,*}_{ij} \right) = d_{i} + \underbrace{\bar{\mu}^{A} - \bar{\mu}^{B}}_{\text{bias}}.  $$

The bias term, $\bar {\mu }^{A} - \bar {\mu }^{B}$, is not zero if the differentiation pattern is not balanced, namely the average effect of up-regulation is not *exactly* equal that of down-regulation for all genes. Other normalization procedures, such as the quantile normalization and rank normalization, also introduce certain bias in such situation, although the mathematical derivation is more cumbersome. Interested readers can find more details in [7, 8].

### Super-delta

The algorithm of the proposed method, super-delta, can be described as follows.

1. The *δ* step. We take the difference between a given gene with all other genes. The difference between the *i*th and *i*′th gene of the *j*th sample in group *a* is denoted by $\delta ^{a}_{ii{\prime },j}$ and has the following representation based on Eq. (3)
  7 $$  \delta_{ii^{\prime},j}^{a} = \mu^{a}_{i} -\mu^{a}_{i^{\prime}} +\epsilon_{ij} -\epsilon_{i^{\prime}j} = x^{a}_{ij} - x^{a}_{i{\prime}j}.  $$
  In a sense, $\delta _{ii{\prime },\cdot }^{a}$ can be considered as the *i*th gene expression normalized by the candidate “house-keeping” gene *i* ^′^. Apparently, $\delta _{ii{\prime },j}^{a}$ is free of sample-specific noise and has variance 2*σ* ^2^.
2. The test step. We compute the two-sample *t*-statistic for all *i* and *i*′ from $\delta _{ii{\prime },j}^{a}$
  8 $$  t_{ii{\prime}} := \frac{\bar{\delta}_{ii{\prime}}^{A} - \bar{\delta}_{ii{\prime}}^{B}}{s_{ii{\prime}}^{p}\sqrt{\frac{1}{N_{A}} + \frac{1}{N_{B}}}},  $$
  9 $$  s_{ii{\prime}}^{p} := \sqrt{\frac{\sum_{j=1}^{N_{A}} \left(\delta_{ii{\prime},j}^{A}-\bar{\delta}_{ii{\prime}}^{A}\right)^{2} + \sum_{j{\prime}=1}^{N_{B}} \left(\delta_{ii{\prime},j{\prime}}^{B}-\bar{\delta}_{ii{\prime}}^{B}\right)^{2}}{N - 2}}.  $$
  Here $s_{ii{\prime }}^{p}$ is the pooled estimate of standard deviation computed from $\delta _{ii{\prime },j}^{a}$ from groups *A* and *B*. *N*=*N*_*A*_+*N*_*B*_ is the total sample size.
3. The summary step. After the above steps, for each gene *i*, we obtain an (*m*−1)-dimensional vector (denoted by **t**_*i*_) of summary statistics *t*_*ii*′_, for *i*′≠*i*. We now need to find a unique representative summary statistic out of them and calculate a single *p*-value for gene *i*. A robust median fold trim median (**MFTM**) estimator is used for this purpose. Specifically, we first remove a proportion (e.g., 20%) of *t*_*ii*′_ that has the largest absolute values from **t**_*i*_. Denote the trimmed vector of *t*-statistics as $\mathbf {t}^{\text {trim}}_{i}$, we define the representative statistic for the *i*th gene as $\sqrt {2}$ times the sample median of $\mathbf {t}^{\text {trim}}_{i}$
  10 $$  t^{\text{MFTM}}_{i} := \sqrt{2} \times \text{Med}\left(\mathbf{t}^{\text{trim}}_{i}\right).  $$
  For comparison, we also implemented methods that uses $\sqrt {2} \bar {\mathbf {t}}_{i}$ (super-delta with untrimmed mean estimator) and $\sqrt {2} \text {Med} ({\mathbf {t}}_{i})$ (super-delta with untrimmed median estimator) as the representative statistic in simulation studies. The use of adjusting factor $\sqrt {2}$ will be explained later.
4. As a “bonus”, we can identify the gene that achieves the median of $\mathbf {t}^{\text {trim}}_{i}$ and call it the *pairing gene* of gene *i*. This pairing gene can be considered as the *empirical* housekeeping gene specific to gene *i*. Note that we have to randomly select one such gene out of two candidates when the size of $\mathbf {t}^{\text {trim}}_{i}$ is even.
5. Calculate raw *p*-values from the representative statistics. Apply a suitable multiple testing procedure to control for overall type I error rate. A gene is declared as DE if its adjusted *p*-value is less than a given threshold such as 0.05.

Heuristically speaking, $t^{\text {MFTM}}_{i}$ produced by super- delta is meant to be an approximation of the *oracle* *t*-statistic, which is the two-sample *t*-statistic computed from the oracle expressions as follows

11 $$  t_{i}^{*} := \frac{\bar{x}^{A} - \bar{x}^{B}}{\sigma_{i}^{p}\sqrt{\frac{1}{N_{A}} + \frac{1}{N_{B}}}},  $$

12 $$  \sigma_{i}^{p} := \sqrt{\frac{\sum_{j=1}^{N_{A}} \left(x_{ij}^{A} -\bar{x}^{A}\right)^{2} + \sum_{j{\prime}=1}^{N_{B}} \left(x_{ij'}^{B}-\bar{x}^{B}\right)^{2}}{N - 2}}.  $$

Here *s* ^*A*^ and *s* ^*B*^ are the two group standard deviations calculated from oracle expressions $x_{ij}^{A}$ and $x_{ij}^{B}$ respectively. Note that the variance of $x^{a}_{ij}$ is *σ* ^2^ but the variance of $\delta _{ii{\prime },j}^{a}$ is 2*σ* ^2^, so we need to multiply the sample median by adjusting factor $\sqrt {2}$. In reality, $x^{a}_{ij}$ is always confounded by $\alpha _{j}^{a}$, so we need to remove this sample-specific noise by subtraction.

The following theorem says that, both the mean and median of the *t*-statistics obtained from a set of non DE genes converge to a multiple of the oracle statistic $t_{i}^{*}$ under the assumption of interchangeable covariance structure.

#### **Theorem 1**

Assume that $\sigma _{i}^{2} \equiv \sigma ^{2}$ for all *i* (interchangeable covariance structure). The conditional mean and median of *t*_*ik*_, for $k\in \mathcal {S}^{0}$, the set of non DE genes, have the following asymptotic representation.

13 $$\begin{array}{*{20}l} E (t_{ik} | \epsilon_{i\cdot}) \stackrel{\mathcal{P}_{\epsilon_{i\cdot}}} {\longrightarrow} \sqrt{\frac{1}{2}} \cdot t^{*}_{i} + O\left(N^{-1}\right), \end{array} $$

14 $$\begin{array}{*{20}l} \text{Med} (t_{ik} | \epsilon_{i\cdot}) \stackrel{\mathcal{P}_{\epsilon_{i\cdot}}} {\longrightarrow} \sqrt{\frac{1}{2}} \cdot t^{*}_{i} + O\left(N^{-1}\right). \end{array} $$

Here $\mathcal {P}_{\epsilon _{i\cdot }}$ stands for convergence in probability.

This theorem justifies the usage of multiplication coefficient $\sqrt {2}$ above. For details of its proof please see Additional file 1.

**Table 1:** A summary table of high-frequency pairing genes

Times being paired	29	27	26	25	24	23	22	21
Number of genes	2	1	1	1	2	1	4	4
Times being paired	20	19	18	17	16	15	14	13
Number of genes	7	4	9	9	19	20	21	18
Times being paired	12	11	10	9	8	7	6	5
Number of genes	25	33	49	60	67	91	127	168

It is reasonable to assume that only a small fraction of all genes are true DE genes. The median fold trim is designed to remove a small subset of very extreme *t*-statistics that are likely the results of pairing with a true DE gene. This trimming procedure does not change the results much when the up- and down-regulated genes are approximately equal; but in case they are highly unbalanced, it can greatly reduce the bias that may be produced by borrowing information from the DE genes, which in turn improves both type I error control and statistical power significantly, as is evident in Table 2. Theoretical discussions of the impact of MFTM can be found in Additional file 1.

**Table 2:** A summary table of three simulation scenarios

	Classical					Super-delta		
	Oracle	Global	medIQR	Quantile	Cyclic-loess	Mean	Median	MFTM
SIM1								
Power	88.82(1.02)	88.17(1.08)	87.84(0.99)	87.71(1.00)	87.61(1.01)	88.48(1.04)	88.89(1.13)	88.85(1.00)
Type I error	0.46(0.07)	0.49(0.08)	0.50(0.07)	0.51(0.08)	0.52(0.07)	0.44(0.08)	0.41(0.08)	0.40(0.07)
SIM2								
Power	92.11(0.81)	90.94(0.89)	90.37(0.95)	90.17(0.93)	89.94(0.95)	91.26(1.01)	91.79(0.94)	92.09(0.77)
Type I error	0.93(0.14)	1.08(0.17)	1.25(0.22)	1.26(0.19)	1.36(0.23)	1.03(0.22)	0.85(0.15)	0.83(0.15)
SIM3								
Power	89.18(1.49)	76.67(1.51)	77.28(1.84)	76.20(1.63)	76.51(1.59)	77.16(1.89)	86.05(1.70)	88.55(1.62)
Type I error	0.61(0.11)	1.53(0.19)	1.42(0.20)	1.55(0.20)	1.52(0.20)	1.46(0.26)	0.61(0.14)	0.54(0.12)

Sample size is 50 for both groups. All *p*-values are **Benjamini-Hochberg** adjusted; **Power**: Approximate statistical power; **Type I error**: Approximate Type I error rate. All these measurements are calculated by averaging over **50** replicates. Numbers within parentheses are standard deviations. All numbers are pertentage rates

## Results

### Differential expression analysis for the real data

**Fig. 1:**
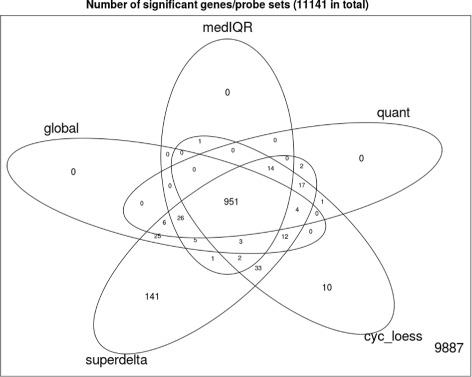
Venn diagram of significant genes (numbers in figure are numbers of genes)

A *t*-test with Welch approximation [26] was implemented after each normalization procedure. All *p*-values were adjusted by the Benjamini-Hochberg procedure [27] to control for false discovery rate (FDR). A gene is selected as differentially expressed if the adjusted *p*-value is less than 0.05 and the fold change [28–30] was greater than 1.25. Numbers of significant DE genes are illustrated in the Venn diagram in Fig. 1. We see that while the number of DE genes selected by the three competing procedures are close, super-delta can detect approximately 15% more significant DE genes (Additional file 2).

### Gene annotation and gene set enrichment analysis

After obtaining the significant gene lists from all procedures, we sorted each list by the absolute values of *t*-statistics. We then annotate the top 30 most significant genes in each DE gene list in order to understand their biological functions. By and large, the majority of these most significant genes identified by all five methods are directly or indirectly related to the formation, development, metastasis, and resistance to chemotherapy of breast cancer. As an example, MCM6 is the most significant gene selected by super-delta method. This gene is a highly conserved mini-chromosome maintenance protein that is essential for the initiation of eukaryotic genome replication. It is known to be a predictive biomarker for breast cancer classification and prognosis [31]. Other notable breast cancer related genes identified by super-delta include YBX1, KPNA2, SKP2, and NAT1. The full lists of significant genes selected by different methods and their detailed annotations can be found in Additional files 2 and 3.

While most pairing genes are related to either basic biological processes or immune responses (see “Further investigations of pairing genesFurtherinvestigations of pairing genes” section for more details), a few interesting exceptions do exist. One such example is ST6GAL1, which is the pairing gene for MCM6. This gene encodes a type II membrane protein that is a member of the glycosyltransferase family, is known to be a breast cancer biomarker that is associated with carcinoma differentiation [32], drug resistance [33], and tumor-associated carbohydrate antigens (TACA) in breast cancer [34]. Given the fact that the mean expression level of ST6GAL1 is high in both the pCR and RD groups and is not differentially expressed, this gene is likely to be active in both subgroups. It has a (super-delta based) t-statistic of 1.347 and adjusted p-value of 0.274. A recent study [35] revealed that CD8+ T cells derived from normal donors are capable of recognizing TACA expressed in carcinomas and responded with high efficiency to glycopeptides in vitro, which has the potential for the design of vaccines for cancer prevention.

Next, we conduct functional enrichment analysis (also known as gene set enrichment analysis, GSEA) using Database for Annotation, Visualization and Integrated Discovery (DAVID version 6.7, [36]) with a focus on KEGG pathways [37]. There are 14 significant KEGG pathways selected by super-delta; three other methods (global, quantile, medIQR) identified 13 significant pathways and loess identified 16 pathways. The first impression of these results are the similarities between them: 12 pathways are identified by all five methods. Almost all of them, such as mismatch repair (KEGG hsa03430), PPAR signaling (KEGG hsa03320), P53 signaling (KEGG hsa04115), Glycolysis/Gluconeogenesis (KEGG hsa00010), are known to be associated with cancer. We would also like to point out that bladder cancer and small cell lung cancer pathways were also significant for all five methods, largely because generic oncogenes such as HRAS and FGFR3, and generic tumor suppressors such as p14ARF, p53, and Rb are identified.

Twopathways are uniquely significant for super- delta: Biosynthesis of unsaturated fatty acids (KEGG hsa01040) and Type II diabetes mellitus (KEGG hsa04930). Unsaturated fatty acids are known to stimulate the proliferation of human MDA-MB-231 breast cancer cells [38–40], and there is a strong link between diabetes mellitus and the risk of breast cancer [41–43].

Compared to super-delta, other methods also identified some unique significant pathways: Pathogenic *Escherichia coli* infection pathway (KEGG hsa05130, by quantile and loess); Valine, leucine and isoleucine degradation pathway (KEGG hsa00280, by both global and medIQR). Both of them seem to have only weak and indirect link to breast cancer. Calcium signaling pathway (KEGG hsa04020) and Pathways in cancer (KEGG hsa05200) are identified only by loess. While Pathways in cancer is apparently related to breast cancer, Calcium signaling pathway represents an ubiquitous cellular activity that is not necessarily induced by cancer. Detailed results of GSEA can be found in Additional file 4.

### Further investigations of pairing genes

As mentioned previously, pairing genes play an important role in super-delta procedure. A pairing gene can be considered as the empirically defined *best* house-keeping gene of a particular gene. Here “best” means that among all possibilities, normalizing the original gene by its pairing gene can produce an adjusted *t*-statistic that approximates the oracle *t*-statistic the best. Its role is comparable to the mean of all genes in global normalization, the medians and IQR’s in median-IQR normalization, and the average distribution quantiles in quantile normalization. A critical difference is that, a pairing gene is found *uniquely* for each gene in any given range, either all or part of the genes being analysed. Therefore, super-delta does not depend on large number of genes recorded and the reliability of using pairing genes is justified by the asymptotic properties stated in Theorem 1.

One interesting fact is that many genes are paired to multiple genes. In fact, only 3149 genes (28.26*%* of all genes) were selected as pairing genes. Table 1 summarizes the pairing frequency of 743 (6.67*%*) genes that were identified as pairing genes for more than 5 times (we call them “candidate house-keeping genes”, Table 1, Additional file 5). Collectively, they were paired to a total of 6510 (58.43*%*) genes.

Annotations of top pairing genes show that most of them have basic functions that are involved in many biological processes. An example is NDUFS2 (paired 29 times), which encodes a protein that is a subunit of mitochondrial membrane respiratory chain NADH dehydrogenase. GO annotations related to this gene include ubiquitin protein ligase binding, which is a very fundamental biological function that are essential for a variety of biological processes. Other notable examples include FAM8A1 (paired 26 times), MYL12A (paired 25 times), and RRP9 (paired 24 times). Interestingly, we also noticed that a significant subset of pairing genes have a direct or indirect relationship with immunity. Examples in this category include GNAI2 (paired 29 times) and STAT5B (paired 15 times). GNAI2 is a member of the chemokine signaling pathway that is known to affect the organization of lymphocytes and the movement of CD4 T cells in lymphoid organs [44, 45]. STAT5B is a key player in multiple biological processes, including ERBB signaling, chemokine signaling, and JAK-STAT signaling pathways, among others. STAT5B deficiency is linked to immunological aberrations such as allergic diseases, immunodeficiencies, autoimmunities, as well as cancers [46].

We then conducted a functional enrichment analysis based on these top pairing genes and found 11 significant pathways. About half of them are related to basic biological functions, such as Ribosome, Hematopoietic cell lineage, Axon guidance, and Regulation of actin cytoskeleton. The other half are related to immune responses, such as Primary immunodeficiency, Natural killer cell mediated cytotoxicity, Viral myocarditis, Autoimmune thyroid disease, Antigen processing and presentation, Allograft rejection, and Leukocyte transendothelial migration. The full list of these 743 top pairing genes and the 11 significant KEGG pathways associated with them are provided in Additional file 5.

### Rank difference analysis

A rank difference analysis was conducted to help understand what kind of genes is likely to be significant after being processed by a certain normalization procedure.

Inthis analysis, we focused on comparing super-delta with quantile, since it is the most widely used approach among all three normalization procedures. For both approaches, we rank the genes from the most significant to the least significant, based on the descending order of the absolute values of *t*-statistics.

**Fig. 2:**
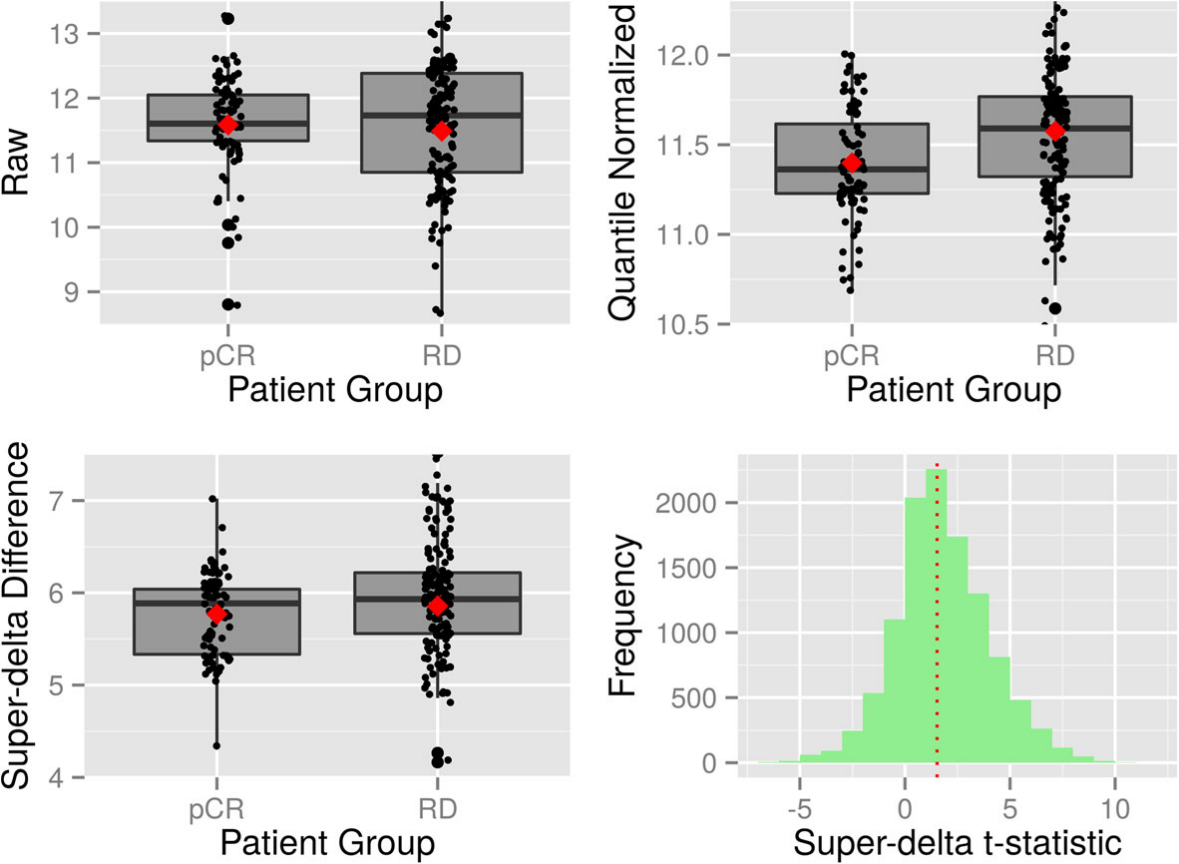
Gene **RPL3** is selected as a DEG by quantile normalization but not by super-delta. Upper left: Parallel boxplot of raw gene expressions; Upper right: Parallel boxplot of quantile normalized gene expressions; Lower left: Parallel boxplot of super-delta differences; Lower right: Histogram of super-delta *t*-statistics by normalizing with all genes. Diamonds on boxplots represent sample means. Dashed vertical line in histogram represents **MFTM**

**Fig. 3:**
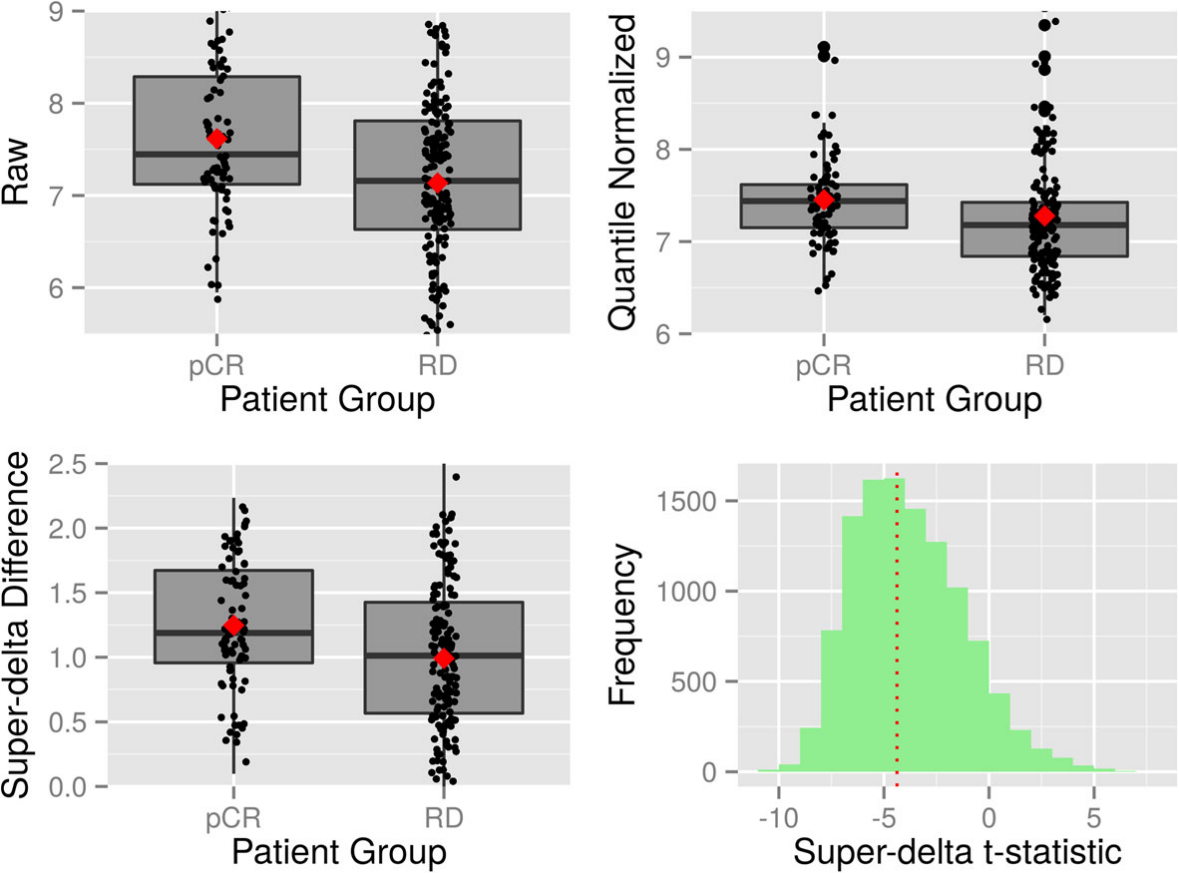
Gene **BIRC5** is selected as DE gene by super-delta but not by quantile normalization. Annotations are the same as Fig. 2

Using the top 1000 most significant genes from one list as reference, we computed the rank differences of these genes with the other list. Large rank difference associated with a gene suggests that this gene is considered much less significant in the second approach. This analysis was performed in both directions. We plotted the original and normalized expression levels of 10 genes with the largest rank differences in both directions in Figs. 2 and 3 and Additional file 6. Judging from the shape of the distribution, it’s clear that super-delta preserves the pattern of raw gene expressions better than quantile. Furthermore, DE genes detected by super-delta have wider separations of the distributions between two groups than those detected by quantile, which suggests that they may have stronger differential expressions than those selected by quantile. One possible explanation is that although quantile is a relatively robust procedure, it is a non-linear transformation that can impose a distortion to the data, especially when the skewness of distribution of two groups differ significantly. The reference quantiles are obtained from an assumed common underlying distribution across all genes and all samples. This assumption is only partially true in reality because not all genes/samples share the common distribution under possibly very different biological conditions. On the other hand, super-delta has a robust trimming method that removes questionable genes from the normalization step, which keeps as much original expression pattern of a gene as possible. For complete information of this rank difference analysis please see Additional file 6.

### Simulation studies

Three related simulation strategies were designed to investigate the power and type I error rate control of all procedures covered in this study. To achieve verisimilitude of the simulation, we estimated all model parameters from the real biological data. Specifically, we estimated the per-sample random effect term variation $\hat {\eta }=0.873$ and the random error term variation $\hat {\sigma }=0.617$ (signal-to-noise ratio is 1.41). These parameters were used for the simulation study. We sorted the absolute values of log2 fold changes and selected the first 1000 (363 up and 637 down regulations) to be considered as true signal (mean group difference). The simulation design is presented as follows.

- Number of genes is 10,000. True signals are 363 up and 637 down regulations.
- Number of genes is 5000. True signals are 363 up and 637 down regulations. Compared with **SIM1**, the only difference is that total number of genes is reduced by a half, which makes the proportion of DE genes doubled.
- Number of genes is 5000. True signals include only the 637 down regulations and all up regulations are removed. Compared with **SIM2**, **SIM3** has a more extreme and unbalanced DE structure.

For each simulation study, three sample sizes (number of slides in each group) were used: **n=50, 75, 100**. One more scenario of unequal sample size ***n***_**1**_ **=50,** ***n***_**2**_ **=100** was also included.

Although the use of MFTM in conjunction with super-delta is recommended, we included two alternative methods, untrimmed mean and median, of selecting representative statistic in super-delta in simulation studies for comparison. Details of these two methods can be found in “Super-delta” section.

Table 2 summarizes results for the case in which the sample size of both groups is *n*=50. It clearly demonstrates that in all cases, super-delta with MFTM is about the same or more powerful than other methods. This advantage is more prominent when the DE structure is strong (SIM2) and/or highly unbalanced (SIM3). More importantly, in all cases, super-delta with MFTM controls type I error rate much better than the other procedures. Overall, the statistical performance of super-delta with MFTM is much closer to that of using the oracle data than all other methods.

Among the other two variants of super-delta, using the untrimmed mean resulted in excessive type I error rate in the most extremely unbalanced case (SIM3); but even in this case, the numbers of false discoveries are slightly smaller than that of the four traditional methods. Overall, the performance of super-delta with the untrimmed median estimator is also very good, with the exception of SIM3, in which case it is unquestionably inferior to super-delta with MFTM but still much better than the four classical methods. Simulation results of other sample size combinations are provided in Additional file 7. Repeating for 50 times also demonstrates the robustness of this simulation design. In the meantime, we also recorded the time consumed by super-delta and quantile normalization. We used the option of automatically selected 1000 baseline genes (which practically would lead to similar results as using all baseline genes) of super-delta and found that super-delta takes about 15- 20% longer time than quantile normalization does. We believe that this small increase of computational cost is acceptable in practice.

## Discussion

Traditional normalization procedures calculate the normalized expression levels in one step by borrowing information from both DE and NDE genes to remove sample-specific variation. Such practice can introduce nontrivial bias when the effects of up- and down-regulated genes to normalization are not exactly the same. In comparison, super-delta first normalizes every gene by all other genes, which generates thousands of normalized values that can help adjust the *t*-statistic for this gene. Because we use subtraction as a means to remove per-sample variation, the *δ* step can be considered as a multivariate extension of the global normalization. In fact, if we take the mean of all *t*_*ii*′_, for *i*′≠*i*, as the representative statistic for the *i*th gene, the results are very similar to that of the global normalization (see columns “global” and “mean” in Table 2). On the other hand, the multivariate nature of the *δ* step enables us to apply a robust trimming algorithm to filter out certain percentages of extremely large or small statistics that are likely to cause bias to the subsequent inference. This filter reduces false discoveries significantly and improves statistical power at the same time when the gene expression pattern is relatively strong and highly unbalanced (SIM3).

It is worth noting that the predecessor of super-delta,the original delta-sequence method (henceforth denoted as delta-seq) had poorer statistical power and more false positives than the traditional methods in many realistic situations [7, 8]. So why does super-delta perform much better? We believe the reason is twofold. First, delta-seq was designed to be a method to select significant *gene pairs*, not individual significant genes [11]. It relies on an imperfect ad hoc method to “break the pairs” and identify significant genes from significant gene pairs [10, 12]. Unlike delta-seq, super-delta is designed to identify significant genes, not gene pairs. It does not rely on the pair-breaking method that leads to excessive type I error. Second, we conduct theoretical derivations to justify the use of coefficient $\sqrt {2}$ in the adjusted *t*-test, which greatly enhances the statistical power of super-delta. As a matter of fact, in most simulation studies, the performance of super-delta in terms of power/type I error rate is very close to the “oracle” method and is much better than its competitors.

In real data analysis, we find that super-delta is largely consistent with other methods (in terms of large proportion of common DEGs) but is slightly more powerful. Functional enrichment analyses reveals that all four methods identified similar biological processes; and super-delta selects two unique pathways that are relevant to breast cancer (Additional file 4).

One under-appreciated but important advantage which super-delta inherited from delta-seq is that we can identify pairing genes (a.k.a. empirical house-keeping genes) by data-driven methods. Biological data analysis shows that these pairing genes either have very broad biological functions thus are good candidate for house-keeping genes; or they play direct or indirect roles in immunity. Further investigations are needed to fully understand the interplay between the main DEGs and those immunity-related pairing genes. A related advantage is that like delta-seq, super-delta is a “local” normalization method which makes it especially suitable for real-world applications.

Imagine that for the reason of saving the cost and processing time, we are only allowed to use a handful of top DE genes as biomarkers in a commercialized *portable* diagnostic device with very limited computational power. We will not be able to faithfully reproduce the differential expression results as defined by the traditional normalization methods because we don’t have enough genes to calculate per-sample mean or median accurately, much less a “reference quantile curve”. Commercial diagnostic devices based on gene expression biomarkers usually rely on polymerase chain reaction (PCR) based platforms, which are more economic and convenient, to measure the expressions for a very small set of pre-specified genes. Biomarker discoveries using traditional normalization methods on microarray or next generation sequencing data cannot be directly translated into these PCR based platforms because the same normalization procedure cannot be performed on PCR platforms. This may be an important reason that accounts for the poor success rate when these biomarkers were tested clinically. On the other hand, for super-delta, top *p*_1_ DE genes need at most *p*_1_ pairing genes in the *δ* step. Empirical evidences show that most pairing genes are reused by more than one other gene (see “Further investigations of pairing genes” section), so the actual number of pairing genes needed to reproduce the results obtained from super-delta faithfully may be even less than *p*_1_. While thorough investigations in a prospective study are required to understand the full impact of pre-processing procedures to predictive models based on gene expressions, we conducted a “proof of concept” simulation study described as follows. We generated an independent training set and test set based on **SIM1** (10,000 genes in total, 1000 true signals, marginally unbalanced DE structure). Sample size are *n*=50 for both groups. Quantile normalization and super-delta + MFTM are compared. We randomly select {10, 20, 50, 100} genes from the top 1,000 genes returned by DE analysis in the training set as features to build a support vector machine (SVM) classifier, and then apply it to the test set. To be fair, for super-delta, we only select half number of genes with their pairing genes and use their differences (deltas) as predictors. The whole process is repeated 50 times. Mean and standard deviation of prediction accuracy are recorded. Quantile has slightly higher prediction accuracy when only 10 genes (compared with 5 deltas) are used. But it is surpassed by super-delta when 20 genes are used. With 50 and 100 genes, advantage of super-delta becomes more prominent in terms of higher accuracy and smaller variability. When most significant genes ranked by *p*-values are used as features, both procedures produce near perfect classifications, and super-delta is noticeably better than Quantile in all four cases. Details of this simulation study can be found in Additional file 7.

We believe super-delta can be easily extended to solve other inferential problems such as one-way ANOVA and linear regression. All we need to do is to prove similar asymptotic properties as in Theorem 1 for those problems. Another extension to super-delta is also possible but may need much more investigation: to create a multivariate-version of the quantile normalization. However, it is not obvious to map the quantile normalization to a local computation just between two genes. One possible solution is to use a first step quantile normalization as a rough guide and then use either weighted linear combination or even a nonlinear transformation for local normalization. A thorough theoretical and empirical study in this direction could be very rewarding in the future.

Finally, we would like to discuss the applicability of super-delta to expression data generated by RNA-seq technology [47, 48]. Although raw RNA-seq reads are *discrete* random variables that are generally modeled by non-normal distributions such as negative binomial distribution [49, 50], it is a common practice to apply non-specific filtering to remove genes with very low reads and then use log-transformation to stabilize variance. These pre-processing steps reduce the granularity of the RNA-seq data and make the distribution much more normal. In fact, some recent comparative studies [51–53] showed that differential expression analysis tools designed for continuous data can achieve comparable, sometimes even slightly better, performance than those based on discrete models. Based on these considerations, we believe that with appropriate adaptations, super-delta can be made applicable for pre-processed RNA-seq data. That being said, a thorough investigation in this direction would be an interesting topic for a future comparative study.

## Conclusions

In summary, we proposed a differential gene expression analysis pipeline that consists of a multivariate extension of the global normalization method (the *δ* step) to remove sample-specific variation; an adjusted two sample Welch *t*-test (the test step) that takes the variation of both genes of interest and their pairs into consideration; and a robust trimming algorithm (the summarizing step) to select one overall statistic to represent the empirical distribution of *δ*s pertain to every gene. Once these representative statistics $\left (t^{\text {MFTM}}_{i}\right)$ are calculated, unadjusted and adjusted *p*-values can be obtained by standard inferential practice. As a new pipeline, super-delta provides new insights to the area of differential gene expression analysis. Solid theoretical foundation supports its asymptotic unbiasedness and technical noise-free properties. Implementation on real and simulated datasets demonstrates its decent performance compared with state-of-art procedures. It also has the potential of expansion to be incorporated with other data type and/or more general between-group comparison problems.

## Additional files

Additional file 1 Theoretical proofs and justifications. This file contains a series of theorem/lemma/proposition/corollary proofs that form the theoretical foundation of super-delta method. (PDF 236 kb)

Additional file 2 Full significant gene lists. This file lists all the significant genes detected by all five methods, each list in a separate worksheet. (XLSX 223 kb)

Additional file 3 Top 30 significant genes’ annotation. This file includes biological annotation of the 30 most significant genes, detected by each method, sorted by the magnitude of *t*-statistics. (XLSX 39 kb)

Additional file 4 Gene set enrichment analysis. This file lists all the significant KEGG pathways obtained by the significant gene lists in Additional file 1. This gene set enrichment analysis was implemented in DAVID. (XLSX 15 kb)

Additional file 5 A Comprehensive investigation of pairing genes. This file contains full information of the pairing genes of super-delta, including the gene set enrichment results of the pairing gene list of the significant genes in Additional file 1. (XLSX 152 kb)

Additional file 6 Rank difference analysis. This file contains information of most differently ranked significant genes between super-delta and quantile normalization. (XLSX 852 kb)

Additional file 7 Simulation study. This file contains the simulation result tables similar to Table 2 of the main text, for all the sample size combinations used. (XLSX 3229 kb)

## Abbreviations

BH: Benjamini-Hochberg
DAVID: The database for annotation, visualization and integrated discovery
DE(G): Differential expression (gene)
FDR: False discovery rate
GEO: Gene expression Omnibus
GO: Gene ontology
GSEA: Gene set enrichment analysis
IQR: Interquartile range
KEGG: Kyoto encyclopedia of genes and genomes
MFTM: Median fold trimmed median
PCR: Polymerase chain reaction
pCR: Pathologic complete response
RD: Residual disease
TACA: Tumor-associated carbohydrate antigens
